# A framework for estimating forest disturbance intensity from successive remotely sensed biomass maps: moving beyond average biomass loss estimates

**DOI:** 10.1186/s13021-015-0039-0

**Published:** 2015-12-02

**Authors:** T. C. Hill, C. M. Ryan, M. Williams

**Affiliations:** 1 Department of Earth and Environmental Science, University of St Andrews, Irvine Building, North Street, St Andrews, UK; 2 School of GeoSciences, The University of Edinburgh, Edinburgh, UK; 3 The NERC National Centre for Earth Observation, St Andrews, UK

**Keywords:** Deforestation, Forest, Degradation, Disturbance, Intensity, Biomass, REDD, Satellite, Remote sensing, Carbon

## Abstract

**Background:**

The success of satellites in mapping deforestation has been invaluable for improving our understanding of the impacts and nature of land cover change and carbon balance. However, current satellite approaches struggle to quantify the intensity of forest disturbance, i.e. whether the average rate of biomass loss for a region arises from heavy disturbance focused in a few locations, or the less severe disturbance of a wider area. The ability to distinguish between these, very different, disturbance regimes remains critical for forest managers and ecologists.

**Results:**

We put forward a framework for describing all intensities of forest disturbance, from deforestation, to widespread low intensity disturbance. By grouping satellite observations into ensembles with a common disturbance regime, the framework is able to mitigate the impacts of poor signal-to-noise ratio that limits current satellite observations. Using an observation system simulation experiment we demonstrate that the framework can be applied to provide estimates of the mean biomass loss rate, as well as distinguish the intensity of the disturbance. The approach is robust despite the large random and systematic errors typical of biomass maps derived from radar. The best accuracies are achieved with ensembles of ≥1600 pixels (≥1 km^2^ with 25 by 25 m pixels).

**Summary:**

The framework we describe provides a novel way to describe and quantify the intensity of forest disturbance, which could help to provide information on the causes of both natural and anthropogenic forest loss—such information is vital for effective forest and climate policy formulation.

**Electronic supplementary material:**

The online version of this article (doi:10.1186/s13021-015-0039-0) contains supplementary material, which is available to authorized users.

## Background

Tropical deforestation has been estimated to occur at a rate of 13 million ha per year [1], with an associated net loss of forest biomass of 1.3 ± 0.7 Pg C year^−1^ [2]. Remote sensing has been successful at mapping global deforestation [3, 4]. However, deforestation presents a simplified view of forest disturbance that ignores the many graduations of lower intensity, but often widespread, forest disturbance and degradation [5]. Forest disturbance refers to the mechanisms which limit biomass by causing its destruction [6]. The impact of forest disturbance is highly variable, leading to the total or partial loss of biomass through a diverse range of natural (e.g. disease, droughts, fires, herbivory and windstorms) and/or anthropogenic processes (e.g. urbanisation, agriculture, selective logging, and fires). Remotely sensed information on the spatial extent and intensity of forest disturbance would be extremely useful for managers and ecologists in attempts to develop a mechanistic understanding of forest degradation and the processes of forest disturbance [7, 8].

Unfortunately, whilst satellites have the coverage to provide global information on forest disturbance, very few studies have attempted to do so [9]. The focus of remote sensing has remained on mapping deforestation (e.g. [3]) and arises from a number of technological limitations which include, amongst other factors, observation precision and the relative “directness” of the observation [10–12]. Ideally remote sensing measurements would have a direct dependence on the quantity being estimated; however current optical satellite derived estimates of forest disturbance are not able to achieve this ideal. Instead, optical satellite measurements of forest disturbance rely on observing physical properties that are expected to correlate with disturbance (e.g. changes in leaf area that can be detected by satellites due to a change in the absorption in the chlorophyll spectral bands). When these correlations change, indirect measures cannot be expected to provide a robust estimate of disturbance.

As Synthetic aperture radar (SAR) is sensitive to the structural properties of forests it can be thought of as a more direct measure of forest biomass than the passive optical alternatives [8, 13, 14]. SAR provides one of the only viable data sources currently available for global monitoring of forest disturbance at moderate spatial resolutions. However, the low signal to noise ratio of SAR leads to poor precision and large uncertainty in the estimated biomass of individual pixels [13] and this equates to high levels of uncertainty when detecting biomass change at the pixel level. Filtering can be used to improve the signal to noise ratio of SAR, but this improvement comes at the expense of a reduction in the effective spatial resolution of the biomass change estimates. Therefore remote sensing can provide regional estimates of biomass change or fine scale maps of deforestation, but it cannot yet be said to truly determine the intensity of forest degradation across scales [13].

In this study we set out a novel framework for quantifying the intensity of forest disturbance from successive remotely sensed biomass maps. This approach can describe both focused high intensity forest loss and low intensity, widespread degradation. We provide an example methodology to exploit this framework using SAR data with realistic observation errors. We explore the strengths and limitations of this approach using an observation system simulation experiment.

## Results and discussion

### A framework for quantifying the intensity of forest disturbance

The framework that we propose describes forest disturbance for an area in which all satellite observation pixels are assumed to experience the same disturbance regime. That is, whilst each pixel might or might not be disturbed, each pixel within each area has the same probability of being disturbed and the same relative loss of biomass when disturbed. We consider an ensemble of *n* remote sensing pixels, which are observed on two successive dates (*t* = 1 and *t* = 2). This approach builds on an earlier framework set out in Williams [15] that used the biomass distribution at a single point in time. The first advantage of this new approach is that it allows ensemble statistics to be calculated, negating the limitations of poor signal to noise of individual biomass pixels by estimating ensemble’s mean fractional loss of biomass per year (*E*_M_). The second advantage of the framework is that it permits *E*_M_ to be split into two factors for the ensemble: the probability of disturbance per year for each ensemble pixel member (*E*_P_) and the fractional loss of biomass per disturbance for each disturbed ensemble pixel member (*E*_I_), Eq. 1.

1 $$E_{\text{M}} \approx E_{\text{P}} E_{\text{I}}$$

Both *E*_M_ and its factors *E*_P_ and *E*_I_ can take values ranging from 0–1. Where, for example, an *E*_P_ = 0.05 would imply a 1 in 20 chance of each pixel being disturbed in a year. An *E*_I_ = 0.2 implies that, if disturbed, a pixel will lose 20 % of its biomass. Combining these example factors would lead to the expectation of *E*_M_ = 0.01, or a 1 % reduction in mean biomass for the ensemble. The ensemble size can be picked to balance the competing requirements of high precision on estimates of *E*_M_, *E*_P_ and *E*_I_ and meeting the assumption of a continuous disturbance regime.

**Fig. 1:**
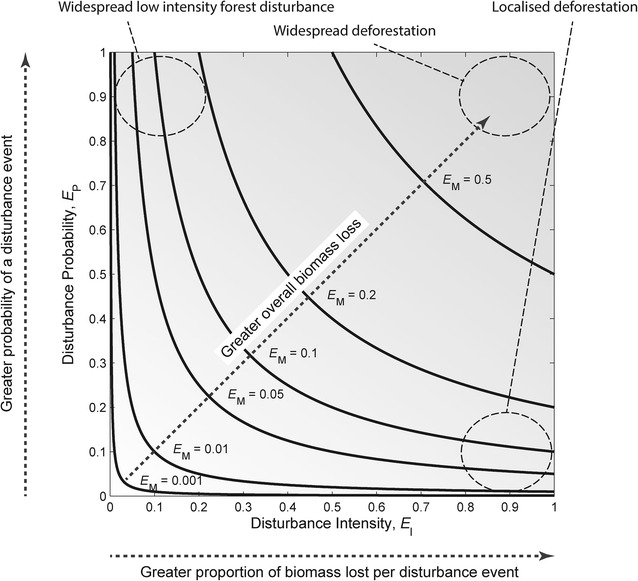
The relationships between the magnitude of disturbance (i.e. the mean fractional disturbance of a region per year, *E*_M_), the disturbance probability (i.e. the probability of a pixel being disturbed each year, *E*_P_), and the disturbance intensity (i.e. the fraction of biomass lost if disturbed, *E*_I_) are shown. Indicated within the figure are regions corresponding to widespread forest degradation, localised deforestation and widespread deforestation

The inclusion of the factors *E*_P_ and *E*_I_ allows a flexible description of biomass loss, without the arbitrary distinction between deforestation and lower intensity forest disturbance. Forest disturbance (other than deforestation) can be represented by the parameter space 0 < *E*_M_ < 1, 0 < *E*_I_ < 1 and 0 < *E*_P_ ≤ 1 (Fig. 1). There are also several special cases: the total deforestation of the ensemble area (*E*_M_ = 1, *E*_I_ = 1 and *E*_P_ = 1); partial deforestation within the area (0 < *E*_M_ < 1, *E*_I_ = 1 and 0 < *E*_P_ < 1); and no disturbance (*E*_M_ = 0).

Describing forest disturbance using this framework has a number of advantages over traditional descriptors as it avoids the need for an arbitrary threshold for a forest cover loss used in other studies, e.g. [16]. In turn, this allows for a more nuanced description of forest disturbance than is possible with categorical land cover classes, or measures of forest cover.

### Using the framework to estimate biomass loss and disturbance intensity

**Fig. 2:**
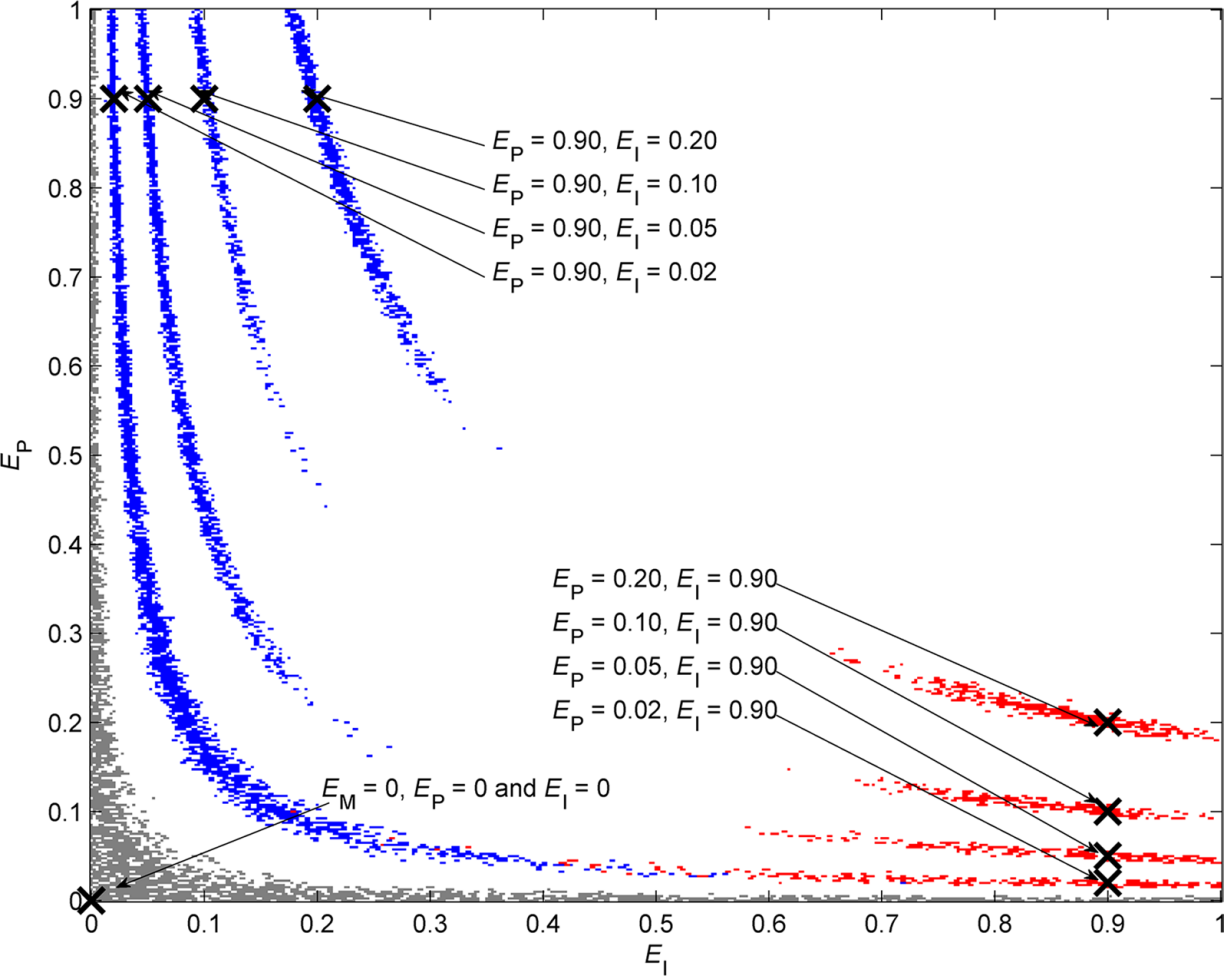
Estimates from the biomass difference approach for nine different combinations of disturbance intensity and disturbance probability. *Crosses* indicate the actual *E*_P_ and *E*_I_ used for each synthetic analysis. *Coloured areas* indicate the most likely combinations of *E*_I_ and *E*_P_ as estimated by the difference approach. The *filled areas* encompass the first 95 % of the cumulative likelihood, *L*. Low intensity cases are shown in *blue*, high intensity cases are shown in *red* and the special case of no disturbance is shown in *grey*

Using the disturbance framework, our observation system simulation experiments (OSSE) show it is possible to robustly estimate the mean biomass loss for the ensemble (*E*_M_) and also the disturbance regime, as described by *E*_P_ and *E*_I_ (Fig. 2; Table 1). *E*_M_ is better constrained than either *E*_I_ or *E*_P_, reflecting the direct impact of *E*_M_ on the mean of the biomass distribution versus the more variable impacts of the *E*_I_ or *E*_P_ on the second and third order moments: standard deviation and skew. With larger mean disturbances the constraints on *E*_I_ or *E*_P_ improve, presumably due to the larger number of pixels effected and/or greater impact on the observed biomass.

**Table 1:** The maximum (97.5 % CI), minimum (2.5 % CI) and 95 % CI range (maximum–minimum) for estimates of *E*_*M*_ *, E*_*I*_ and *E*_*P*_ are shown for each of the test

Figs.	Description	Ensemble size (*n)*	Actual *E*_I_	Actual *E*_P_	Actual *E*_M_	Est. *E*_I_ (max/min/range)	Est. *E*_P_ (max/min/range)	Est. *E*_M_ (max/min/range)
2	High intensity	1600	0.9	0.02	0.018	1.000/0.178/0.823	0.100/0.015/0.085	0.023/0.012/0.011
2	High intensity	1600	0.9	0.05	0.045	1.000/0.580/0.420	0.083/0.043/0.040	0.053/0.040/0.013
2	High intensity	1600	0.9	0.1	0.090	0.993/0.618/0.375	0.148/0.093/0.055	0.097/0.084/0.014
2	High intensity	1600	0.9	0.2	0.180	0.998/0.655/0.343	0.283/0.180/0.103	0.195/0.168/0.027
2	Low intensity	1600	0.02	0.9	0.018	0.720/0.013/0.708	1.000/0.020/0.980	0.023/0.011/0.012
2	Low intensity	1600	0.05	0.9	0.045	0.265/0.040/0.225	1.000/0.163/0.838	0.050/0.038/0.012
2	Low intensity	1600	0.1	0.9	0.090	0.208/0.088/0.120	1.000/0.443/0.558	0.100/0.086/0.014
2	Low intensity	1600	0.2	0.9	0.180	0.363/0.173/0.190	1.000/0.508/0.493	0.188/0.168/0.020
2	No disturbance	1600	0	0	0.000	1.000/0.000/1.000	1.000/0.000/1.000	0.006/0.000/0.006
3	High intensity: Bias = 160 gC m^-2^	1600	0.9	0.05	0.045	1.000/0.653/0.348	0.060/0.038/0.023	0.043/0.035/0.008
3	High intensity: Bias = 0 gC m^-2^	1600	0.9	0.05	0.045	1.000/0.468/0.533	0.098/0.038/0.060	0.049/0.035/0.014
3	High intensity: Bias = −160 gC m^-2^	1600	0.9	0.05	0.045	1.000/0.518/0.483	0.108/0.050/0.058	0.060/0.049/0.011
3	Low intensity: Bias = 160 gC m^-2^	1600	0.05	0.9	0.045	0.188/0.035/0.153	1.000/0.215/0.785	0.044/0.032/0.012
3	Low intensity: Bias = 0 gC m^-2^	1600	0.05	0.9	0.045	0.325/0.040/0.285	1.000/0.148/0.853	0.052/0.039/0.012
3	Low intensity: Bias = −1600 gC m^-2^	1600	0.05	0.9	0.045	0.283/0.048/0.235	1.000/0.173/0.828	0.059/0.046/0.012
4	Area = 2000 by 2000 m	6400	0.05	0.9	0.045	0.128/0.045/0.083	1.000/0.350/0.650	0.048/0.042/0.006
4	Area = 1000 by 1000 m	1600	0.05	0.9	0.045	0.298/0.043/0.255	1.000/0.150/0.850	0.053/0.041/0.013
4	Area = 500 by 500 m	400	0.05	0.9	0.045	0.848/0.040/0.808	1.000/0.045/0.955	0.061/0.033/0.028
4	Area = 250 by 250 m	100	0.05	0.9	0.045	0.980/0.023/0.958	1.000/0.028/0.973	0.074/0.019/0.055

The 95 % CI range is expected encompass the actual *E*_M_, *E*_I_ and *E*_P_ used to generate the synthetic observations

### How the framework is affected by observation bias

**Fig. 3:**
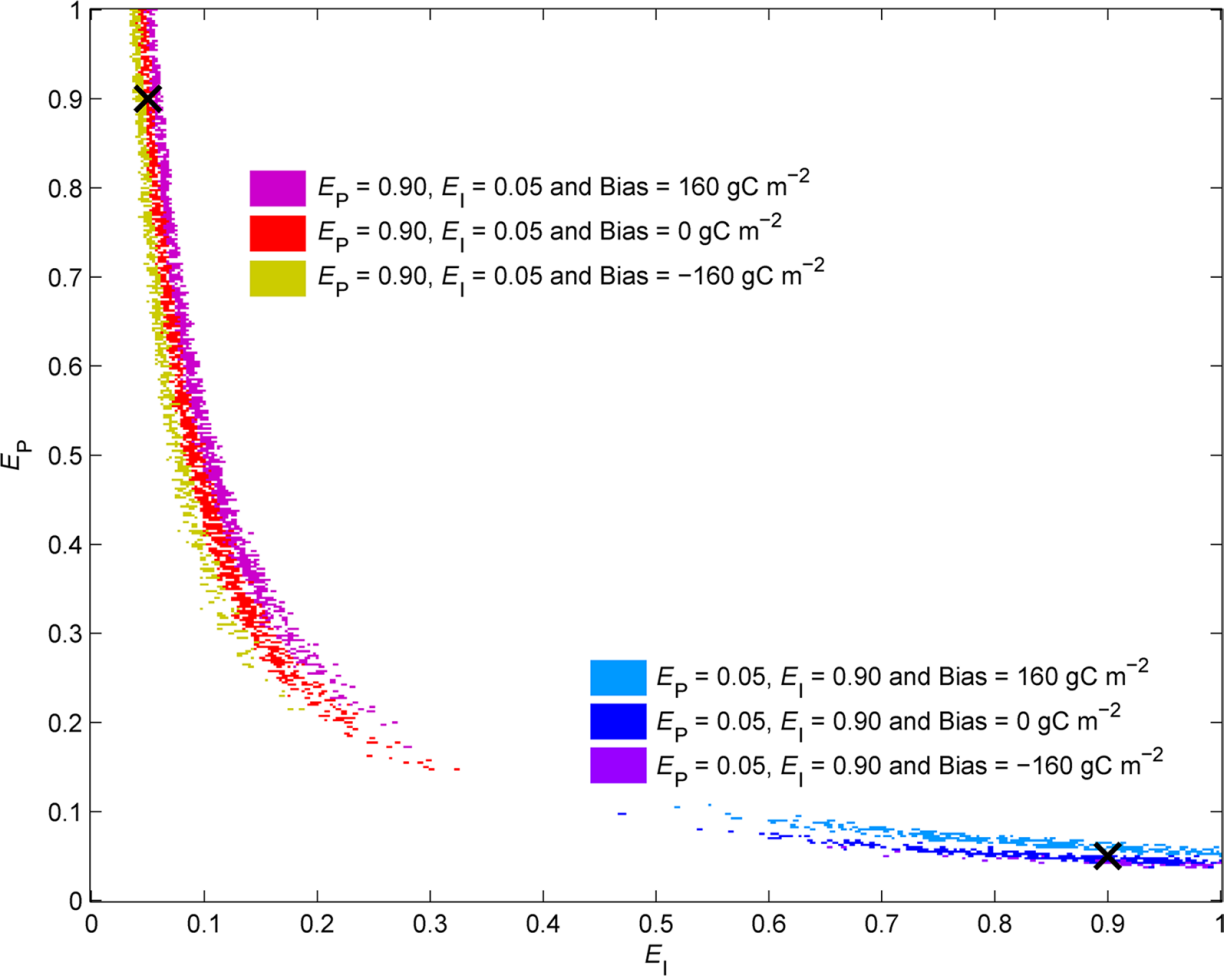
As for Fig. 2, but showing the impact of observation bias on estimated *E*_P_ and *E*_I_. Biases of 160, 0 and −160 gC m^−2^ are applied to the first of the biomass images. For each bias two example cases with the same *E*_M_ are shown: (*1*) High intensity disturbance, *E*_I_ = 0.9 and *E*_P_ = 0.05. (*2*) Low intensity disturbance, *E*_I_ = 0.05 and *E*_P_ = 0.9

It is highly likely that biomass maps used with the framework will not only have random noise, but also systematic observation errors [13], therefore we test the framework’s sensitivity to bias. The inclusion of a realistic observation bias of ±160 gC m^−2^ (±1.6 tC ha^−1^) [13] had the largest impacts on the estimates of *E*_M_, which showed a bias of 0.004 (0.4 %). However *E*_I_ and *E*_P_ were less affected (Fig. 3; Table 1) and it was still possible to distinguish high and low intensity disturbance regimes. The bias in the estimate for *E*_M_ is consistent with change in the mean biomass that is implied by a bias of 160 gC m^−2^, and would therefore apply to any other approach to estimating biomass loss.

### What is the optimal ensemble size?

**Fig. 4:**
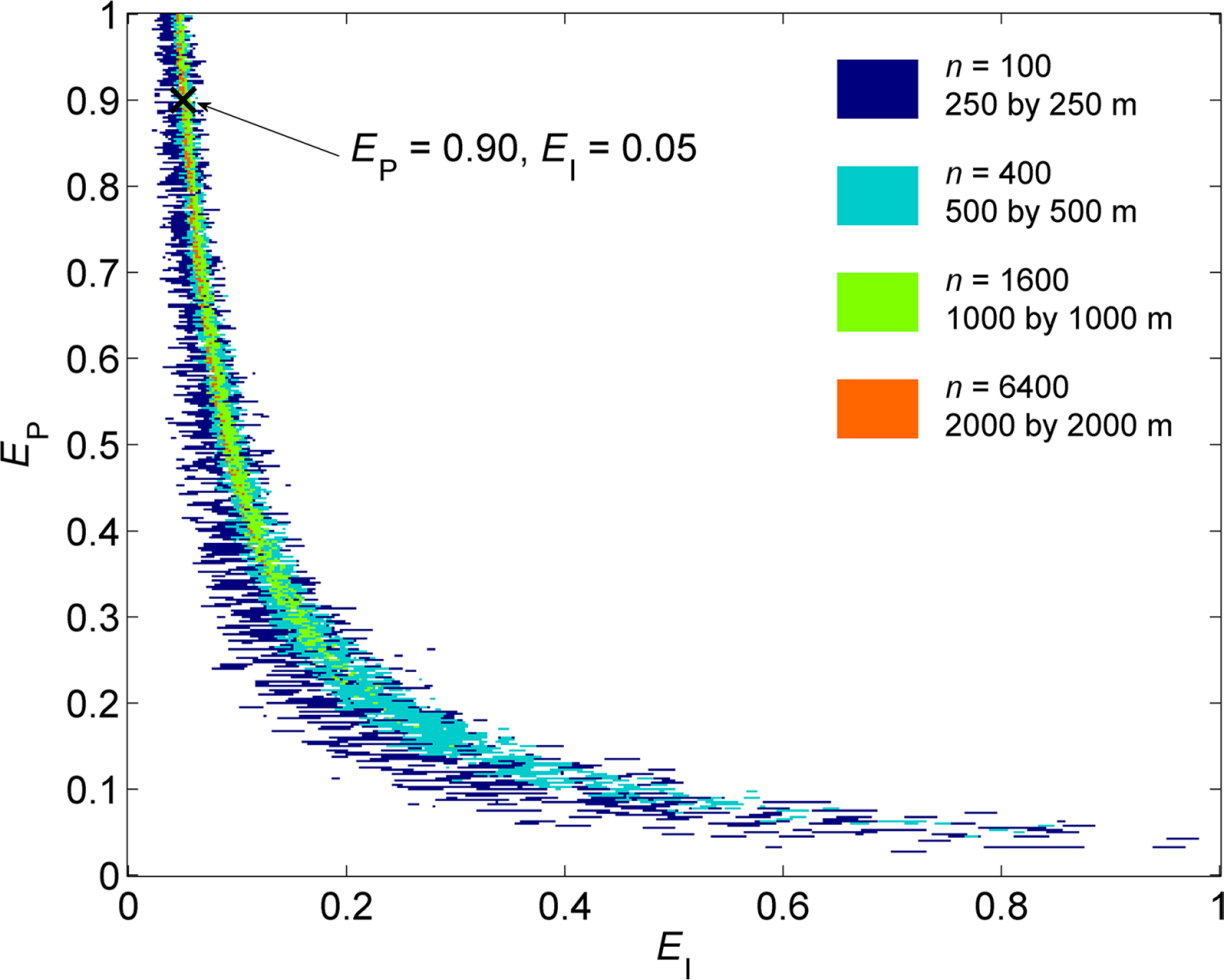
The effect of ensemble size (the number of pixels) on the analysis. The cross shows the synthetic truth and the *coloured areas* indicate the most likely *E*_I_ and *E*_P_

The accuracy of predictions using the framework are best when considering an ensemble size of *n* ≥ 1600, whilst for *n* ≤ 400, the precision of our estimates drops rapidly (Fig. 4; Table 1). Therefore, for the 25 m pixels typical of current SAR biomass estimates, the recommended ensemble size covers an area of at least 1 km^2^ or 100 ha. The implication is that it must be reasonable to assume that the disturbance regime is constant across areas of at least 100 ha. We expect this minimum area to be robust, at least for ALOS PALSAR data, but the sensitivity to ensemble size should checked in each new study. It is worth noting that there is no requirement for these areas to be rectangular, or even contiguous. The grainy texture evident in the estimates from the analyses is due to the simulation of stochastic disturbances (Figs. 2, 3, 4). This graining is reduced with increasing ensemble size, but can be further reduced, at significant computational expense, by averaging repeat runs of the maximum likelihood estimation.

### Alternative formulations of the framework

We formulated the framework in terms of a fractional change in biomass for both *E*_M_ and *E*_I_. This is not the only possible formulation, nor is it necessarily the optimal for all situations; however, it is a mathematically simple approach to maintaining a positive (i.e. plausible) biomass for each pixel. The implicit assumptions of the formulation are: 1) that all pixels are equally likely to be disturbed, and 2) that when disturbed a fixed fraction is lost, irrespective of the starting biomass. It is possible to imagine scenarios that would not be well described by our scheme where (say) selective logging only targets the largest trees (i.e. *E*_P_ is high for pixels above a threshold biomass and zero for all others). These scenarios do not contradict our first assumption of common disturbance regime for the ensemble and it should be possible to restate the parameters used in the framework to accommodate a particular set of assumptions about the disturbance regime. However the inclusion of more complicated mathematical representations and, specifically, more parameters, can be expected to increase the challenge of estimating the parameters of any new framework formulation and decrease its general applicability.

### Limitations to the framework

Finally there are two notable caveats: Firstly, the success of the approach is strongly tied to the ability to characterise the random error of biomass estimates. The design of the analysis mitigates some of the errors as biomass errors that are consistent between the two biomass maps will be removed by this differencing. Whilst Ryan et al. (2012) report normal errors in the biomass domain, other studies assume errors will actually be normal in the dB (i.e. log_10_) domain [17] which would result in log-normal, asymmetric errors on biomass estimates. We therefore reran the synthetic experiments with 0.5 dB error and achieved similar results. The second caveat is that the biomass differencing approach assumes the carbon model (A-DALEC) is unbiased. This assumption is, by definition, valid in a observation system simulation experiment (OSSE). However in practice the productivity of the ecosystem is not known perfectly, and so estimates of *E*_M_ can be expected to be biased. However the assumption is not as crude as it at first might seem: ignoring the production term (as is implicitly done by most deforestation algorithms) makes the assumption that the forest otherwise in steady state. This issue is likely to be more severe in field sites where less information is available on which to provide independent estimate of the rates of aboveground biomass accumulation, e.g. [18].

## Conclusions

From a management and policy perspective it is important to be able to distinguish between the different intensities of biomass loss as they may be associated with different disturbances mechanisms (e.g. low intensity disturbances are likely driven by a need for timber or fuel, and high intensity disturbances driven by a need for agricultural land) [19]. However, current estimates of deforestation and biomass loss are not adequate for estimating lower intensity forest disturbance. Theoretically, high resolution biomass loss estimates could provide fine-scale estimates of forest degradation, but the current precision is not adequate and there is no immediate prospect of this changing, partly due to the speckle and other noise in SAR imagery [20]. The framework that we have described is a pragmatic representation of forest degradation that uses ensemble statistics to mitigate the poor precision of current SAR biomass estimates [13, 17, 20]. SAR is expected to remain a key technique for global mapping of forest biomass, and the framework we propose is compatible with the upcoming BIOMASS and new L-band satellites [14]. Using an OSSE we have shown that is possible to robustly estimate the parameters of the framework to describe forest disturbance, provided that two successive biomass maps, separated by at least one year, are available. We suggest that using a similar framework will allow remote sensing studies to provide more relevant constraints on estimates of land-use change.

## Methods

### Estimating the framework parameters

A number of approaches could be taken to estimating *E*_M_, *E*_I_ and *E*_P_, we chose to use maximum likelihood estimation. We identify the combinations of *E*_I_ and *E*_P_ that allow a simulations to most closely match the observed changes in the biomass distribution from time *t* = 1 to time *t* = 2, for an ensemble of *n* pixels.

The observed biomass (*O*_*i*_^*t*^) of the *i*th pixel at time *t* is sum of the actual biomass (*B*_*i*_^*t*^) and the measurement noise (*N*_*i*_^*t*^), Eq. 2. The observed biomass at time *t* + 1, follows the same logic (Eq. 3). The actual biomasses are related via the growth *G*_*i*_^*t*^ and disturbance *D*_*i*_^*t*^ of each pixel in the ensemble (Eq. 4).

2 $$O_{i}^{t} = B_{i}^{t} + N_{i}^{t} \;\;\,{\text{for }}\;i = \, 1, \, 2, \, 3, \ldots ,n$$

3 $$O_{i}^{t + 1} = B_{i}^{t + 1} + N_{i}^{t + 1} \;\;\,{\text{for}}\;\;i = { 1},{ 2},{ 3}, \ldots ,n$$

4 $$B_{i}^{t + 1} = B_{i}^{t} + G_{i}^{t} - D_{i}^{t} \;\;\,{\text{for}}\;\;i = { 1},{ 2},{ 3}, \ldots ,n$$

Similarly we are able to simulate the observed biomass at time *t* (*Os*_*i*_^*t*^) based on the simulation biomass (*Bs*_*i*_^*t*^) added to the simulated noise (*Ns*_*i*_^*t*^), Eq. 5. The observed biomass at time *t* (*Os*_*i*_^*t*+1^) is calculated from the simulation biomass (*Bs*_*i*_^*t*^) through the sum of the simulated growth (*Gs*_*i*_^*t*^), the simulated disturbance (*Ds*_*i*_^*t*^), and the simulated noise (*Ns*_*i*_^*t*+1^), Eq. 6.

5 $$Os_{i}^{t} = Bs_{i}^{t} + Ns_{i}^{t} \;\;\,{\text{for}}\;\;i = { 1},{ 2},{ 3}, \ldots ,n$$

6 $$Os_{i}^{t + 1} = Bs_{i}^{t} + Gs_{i}^{t} - Ds_{i}^{t} + Ns_{i}^{t + 1} \;\;\,{\text{for}}\;\;i = { 1},{ 2},{ 3}, \ldots ,n$$

where *Ds*_*i*_^*t*^ is related to the disturbance parameters in Eq. 7.

7 $$Ds_{i}^{t} = \left\{ {\begin{array}{*{20}c} 0 & {{\text{if }}U([0,1]) \ge E_{p} } \\ {E_{I} Bs_{i}^{t} } & {otherwise} \\ \end{array} } \right.\;\;{\text{for}}\;\;i = { 1},{ 2},{ 3}, \ldots ,n$$

We model *Gs*_*i*_^*t*^ using the A-DALEC model, a biogeochemical model of carbon cycling in forests, which runs at an annual time step (see Additional file 1 A for full details) [15, 21]. *Ns*_*i*_^*t*^ and *Ns*_*i*_^*t*+1^are based on observed pixel uncertainty in SAR biomass estimates [13]. We cannot base *Bs*_*i*_^*t*^ directly on the observed biomass *O*_*i*_^*t*^, as the observation noise *N*_*i*_^*t*^ cannot be determined (and thus removed) for each pixel, and so the A-DALEC model is used to spin-up an estimate of *Bs*_*i*_^*t*^, see Additional file 1 section B for full details. The equations above assume that *t* = 1 and *t* = 2 are separated by 1 year. When the separation is more than 1 year, as in the case of our OSSE, the base *Bs*_*i*_^*t*^ should have the annual growth and disturbance applied once for each year of separation.

95 % confidence intervals for the factors *E*_P_ and *E*_I_ are found using a maximum likelihood estimation routine, see Additional file 1 C for full details. This routine finds the most likely combinations of *E*_P_ and *E*_I_ based on minimising the differences between the observed and simulated ensemble distributions; Δ*O* = *O* ^*t*+1^ − *O* ^*t*^ and Δ*Os* = *Os* ^*t*+1^ − *Os* ^*t*^.

### Testing the framework parameter estimates

To allow the technique to be assessed against a known ‘truth’ we use a set of observation system simulation experiment (OSSE). In the OSSE we simulate two successive (noisy) biomass observations which have observation noise and a definable disturbance regime applied. The OSSE observations are derived by spinning up the A-DALEC model to match ALOS observations within the Gorongosa and Nhamatanda districts of Sofala province in central Mozambique. The area is dominated by dry miombo woodland, the dominant woodland type in Southern Africa, see Additional file 1 B. The first OSSE observation, *t* = *1*, is taken at the end of the 1st year, the second observation, *t* = *2*, is taken at the end of the 5th year, i.e. separated by 4 years. Separate spin-ups are performed for the Δ*O* and Δ*S* simulations. Three tests are performed; the first to assess the ability of the approach to identify *E*_M_, *E*_I_ and *E*_P_, the second to assess the impact of observation bias and the third to determine impact of ensemble size.

### Determining the accuracy of the framework parameter estimates: test 1

A set of nine analyses were used to test the ability of our approach to identify *E*_M_, *E*_I_ and *E*_P_. Each analysis was performed for the same size ensemble of *n* = 1600 pixels, which equates to an area of 1000 by 1000 m given the 25 by 25 m pixel size of the ALOS biomass maps [13]. We perform OSSEs with four levels of mean disturbance *E*_M_ = 0, 0.018, 0.045, 0.090 and 0.180. These disturbance fractions equate to annual biomass loss rates of 0, 1.8, 4.5, 9 and 18 %. For each nonzero *E*_M_ we simulate a high intensity (*E*_I_ ≫ *E*_P_) and low intensity (*E*_I_ ≪ *E*_P_) disturbance scenario. In the high intensity cases only a few pixels are disturbed, but when disturbed 90 % of the biomass is lost (i.e. *E*_I_ = 0.9). In the low intensity cases, 90 % of the pixels are disturbed (i.e. *E*_P_ = 0.9), but each disturbance results in a small loss of biomass at the pixel level. The various combinations of factors are shown in Table 1.

### Determining the impact of observation bias on the framework parameter estimates: test 2

To simulate the impact of observation bias we perturb the synthetic experiments using the bias observed in the actual biomass maps [13]. Only the first biomass observation (i.e. at time *t* = 1) is biased by −160, 0, and 160 gC m^−2^. The impact of the bias is assessed using both a low intensity case (i.e. *E*_I_ = 0.05 and *E*_P_ = 0.9), and a high intensity case (i.e. *E*_I_ = 0.9 and *E*_P_ = 0.05). In both cases the OSSE has an *E*_M_ = 0.045 and an ensemble size of *n* = 1600 pixels.

### Determining the impact of ensemble size on the framework parameter estimates: test 3

The impact of varying the ensemble size is tested for *n* = 6400, 1600, 400, and 100. These ensembles equate to areas of 2000 by 2000 m (6400 pixels), 1000 by 1000 m (1600 pixels), 500 by 500 m (400 pixels), and 250 by 250 m (100 pixels). The areas are centred on the same location. We analyses these areas for a single low intensity disturbance regime *E*_M_ = 0.045, *E*_I_ = 0.05 and *E*_P_ = 0.9.

## Additional file

**Additional file 1.** A framework for estimating forest disturbance intensity from remotely sensed biomass maps: moving beyond average biomass loss estimates.
