# Supplementary material for: A framework for estimating forest disturbance intensity from successive remotely sensed biomass maps: moving beyond average biomass loss estimates
Source: Carbon Balance Manag. 2015 Dec 2;10:27. doi: 10.1186/s13021-015-0039-0 (PMC4666897; doi:10.1186/s13021-015-0039-0)
Supplement: Supplementary file 1 — Additional file 1. A framework for estimating forest disturbance intensity from remotely sensed biomass maps: moving beyond average biomass loss estimates. [file 13021_2015_39_MOESM1_ESM.docx]

**Additional File: A framework for estimating forest disturbance intensity from remotely sensed biomass maps: moving beyond average biomass loss estimates.**

**A: The A-DALEC ecosystem model.**

We use the Annual DALEC (A-DALEC) model which is a version of the Data Assimilation Linked Ecosystem Carbon (DALEC) model [[1](#_ENREF_1), [2](#_ENREF_2)] which has been modified to run on an annual timestep. The A-DALEC model is calibrated for Miombo woodland using ground inventory data [[1](#_ENREF_1), [3](#_ENREF_3), [4](#_ENREF_4)]. The A-DALEC model simulates ecosystem carbon (C) mass balance and disturbance, and includes both above- and belowground wood carbon pools. Losses from the C pools are specified either as constant turnover rates, or stochastic disturbances. The A-DALEC model is run with a spatial resolution matching the pixel resolution of the ALOS-PALSAR derived biomass map (25 m by 25 m). Each pixel is simulated independently of the other pixels. To capture the natural sub-pixel variability, every pixel contains 50 `patches’, corresponding to the scale of a canopy-dominant tree, each with its own set of carbon pools. The biomass of the simulated pixels is disturbed on an annual basis according to the *E*_P_ and *E*_I_ parameters. A-DALEC can reproduce uncertain biomass observations by adding a prescribed random error to the simulated biomass of the pixels. In this case we add a random uncertainty that matches the error of the ALOS-PALSAR derived maps.

### B: The study area and model spin-up procedure

The study site lies within the Gorongosa and Nhamatanda Districts of Sofala province in central Mozambique. The study area covers 1,160 km^2^ and is dominated by dry miombo woodland, the dominant woodland type in Southern Africa. Miombo has relatively low aboveground carbon stocks and in general does not exceed 5000 gC m^-2^ (50 tC ha^-1^) [[5](#_ENREF_5)]. The overall rate of above ground biomass loss for the region is 2.3 ± 1.5% per year [[3](#_ENREF_3)]. Further details of the study site characteristics and land use can be found in Ryan et al., [[3](#_ENREF_3)].

We use a biomass map from the 23^rd^ of June 2007 which was derived from radar backscatter imagery from the Phased Array L-band Synthetic Aperture Radar sensor aboard the Advanced Land Observing Satellite (ALOS PALSAR) [[6](#_ENREF_6)]. To create this map, aboveground biomass data for 96 biomass plots were regressed against each of the radar backscatter image from ALOS PALSAR [[3](#_ENREF_3)]. The resulting regressions were then used to produce the biomass map for the region. Ryan et al. (2012) describes the generation of this map in detail, which has a pixel size of 25 m with 16 equivalent looks. The ALOS PALSAR derived biomass map has a mean biomass of 1,836 gC m^-2^ (18.4 tC ha^-1^), a mean RMSE error of 980 gC m^-2^ (9.8 tC ha^-1^), and an absolute mean bias of 160 gC m^-2^ (1.6 tC ha^-1^). The original map was much larger than required and so a subset of pixels covering 125 by 125 m to 2000 by 2000 m was used.

The model is spun up to a state that matches that of the ALOS PALSAR-derived biomass map. The ALOS PALSAR-derived biomass map has errors which must be accounted for in the spin up procedure. The spin up procedure used 1000 single pixel A-DALEC simulations. Each of these simulations was run for 200 years, from bare ground, with a different randomly selected *E*_P_ Є [0, 1] and *E*_I_ Є [0, 1] to obtain a pseudo-steady state. The lookup table was generated from the end state of each of the runs with a random number added to match the random errors of the ALOS-PALSAR derived biomass map. To spin up the model, each pixel in the observed biomass map is matched to the closest entry in the lookup table and the corresponding A-DALEC model state used as initialisation.

### C: The details of the maximum likelihood parameter estimation

We aim to identify the combinations of *E*_P_ and *E*_I_ which simulate the changes in pixel biomass ($\Delta Os$) that most likely match the observed change pixel biomass ($\Delta O$) between two time points. $\Delta O$and $\Delta Os$are made up of the *n* ensemble members, where difference for the *i*-th ensemble member, $\Delta O_{i}$and $\Delta Os_{i}$, are calculated on a per ensemble pixel basis, Equation S1 and S2.:

$\Delta O_{i}=O_{i}^{t+1}-O_{i}^{t}$ for *i* = 1, 2, 3, …, *n*  Equation S1

$\Delta Os_{i}=Os_{i}^{t+1}-Os_{i}^{t}$ for *i* = 1, 2, 3, …, *n*  Equation S2.

The similarity of $\Delta O$and $\Delta Os$is calculated using a maximum likelihood estimate based on the mean (*μ*)*,* standard deviation (*σ*) and skew (*γ*) of $\Delta O$and $\Delta Os$. We define a column vector, **o**, containing these descriptive statistics for the observed differences $\Delta O$(Equation S3), and a corresponding vector, **s**, for the simulated differences, $\Delta Os$ (Equation S4):

Equation S3

Equation S4

Where *μ*_o_*,* *σ*_o_ and *γ*_o_ are the mean, standard deviation and skew of $\Delta O$, and *μ*_s_*,* *σ*_s_ and *γ*_s_ are the mean, standard deviation and skew of $\Delta Os$. ^T^ indicates the transpose of the row matrix. The relative multivariate maximum likelihood estimator, *L*, is given by Equation S5.

Equation S5

Where **Σ** is the covariance matrix given by Equations S6 and S7. We assume the distributions to be normal and therefore normalise the covariances by (*N*- 1), where *N* is the number of elements and, in this case, is equal to 3. The overlines indicate sample means.

Equation S6

Equation S7

To identify the most likely combination of *E*_P_ and *E*_I_ to explain the observations, A-DALEC simulates $\Delta Os$for all combinations of *E*_P_ = 0.00, 0.0025, 0.0050, …, 1.00 and *E*_I_ = 0.00, 0.0025, 0.0050, …, 1.00. Then the likelihood, *L*, is calculated for each $\Delta Os$. The most likely combinations of *E*_P_ and *E*_I_ are identified as those that account for the highest 95 % of the likelihoods.

This approach is easy to implement, but incurs moderate computation expense. While $\Delta O$ is calculated just once, for the OSSE, $\Delta Os$is calculated separately for each of the 401^2^ = 160,801 combinations of *E*_P_ and *E*_I_ (i.e.  *E*_P_ = 0.00, 0.0025, 0.0050, …, 1.00 and *E*_I_ = 0.00, 0.0025, 0.0050, …, 1.00.).

**Σ** is estimated from repeated simulations for which the only differences arise from the random nature of the disturbances. Each combination of  *E*_P_ = 0.0, 0.05, 0.10, 0.15, and 0.2, and *E*_I_ = 0.0, 0.05, 0.10, 0.15, and 0.2, is run 100 times i.e. a total of 2,500 runs. The mean of the resulting **Σ**s is used.

**References for the Additional File**

1. Williams M, Hill TC, Ryan CM. Using biomass distributions to determine probability and intensity of tropical forest disturbance. Plant Ecology & Diversity. 2012:1-13. doi:10.1080/17550874.2012.692404.

2. Williams M, Schwarz PA, Law BE, Irvine J, Kurpius MR. An improved analysis of forest carbon dynamics using data assimilation. Global Change Biology. 2005;11(1):89-105.

3. Ryan CM, Hill TC, Woollen E, Ghee C, Mitchard E, Cassells G et al. Quantifying small-scale deforestation and forest degradation in African woodlands using radar imagery. Global Change Biology. 2012;18:243-57. doi:10.1111/j.1365-2486.2011.02551.x.

4. Ryan CM, Williams M. How does fire intensity and frequency affect miombo woodland tree populations and biomass? Ecological Applications. 2011;21(1):48-60.

5. Chidumayo EN. Miombo ecology and management: An introduction. London: IT Publications in association with the Stockholm Environment Institute; 1997.

6. Shimada M, Tadono T, Rosenqvist A. Advanced Land Observing Satellite (ALOS) and Monitoring Global Environmental Change. Proceedings of the IEEE. 2010;98(5):780-99. doi:10.1109/Jproc.2009.2033724.
